# Cannabis for Neuropathic Pain in Multiple Sclerosis—High Expectations, Poor Data

**DOI:** 10.3389/fphar.2019.01239

**Published:** 2019-10-22

**Authors:** Thorsten Rudroff

**Affiliations:** ^1^Department of Health and Human Physiology, University of Iowa, Iowa City, IA, United States; ^2^Department of Neurology, University of Iowa Hospitals and Clinics, Iowa City, IA, United States

**Keywords:** Multiple sclerosis, Cannabis, thc, cbd, neuropathic pain

## Introduction

Pain affects around two-thirds of people with multiple sclerosis (PwMS) (Amatya et al., 2018). MS-related pain includes headache (43%), neuropathic pain in the arms or legs (26%), back pain (20%), painful spasms (15%), and trigeminal neuralgia (3.8%) (Foley et al., 2013). The prevalence of neuropathic pain in PwMS, which arises from peripheral or central nerve injury (described by sufferers as the “most terrible of all tortures which a nerve wound may inflict”), dramatically reduces the quality of life of PwMS (Kenner et al., 2007; Jaggi and Singh, 2011). The point prevalence of neuropathic pain in PwMS is nearly 50%, and approximately 75% of patients report having had pain within one month of assessment (O’Connor et al., 2008). Pharmacological treatment in MS-related neuropathic pain principally consists of the use of tricyclic antidepressants, antiepileptic medications, baclofen, anesthetics, and antiarrhythmic agents. However, these treatments are usually unsatisfactory and often have severe side effects (Solaro et al., 2007). Importantly, inadequate neuropathic pain therapy is one of the contributors to the opioid crisis in the US, and therefore, a need for alternative methods of neuropathic pain relief in PwMS is critical (Rummans et al., 2018).

Many anecdotal reports suggest that cannabis and its major cannabinoid components have beneficial effects on pain, particularly neuropathic pain, in PwMS. However, little scientific evidence supports these anecdotes. Many reviews (Zhornitsky and Potvin, 2012; Jawahar et al., 2013; Koppel et al., 2014; Whiting et al., 2015) agree that cannabis might have a positive effect on pain in MS. Unfortunately, most (overview in Nielsen et al., 2018) did not address issues of trial quality and included different drugs, doses, durations, conditions, and outcomes. Thus, several issues regarding cannabis use to treat neuropathic pain in PwMS remain unresolved.

## Medicinal Cannabis—Use, Formulation, and Administration

The term “cannabis” is often used in media and politics and encompasses all drugs based on the *cannabis sativa* plant and its hundreds of compounds, like delta-9-tetrahydrocannabinol (THC), hemp-extracted cannabidiol (CBD), and THC analogues. THC exhibits psychoactive effects, may induce acute psychosis, and impacts executive function (Colizzi and Bhattacharyya, 2017). CBD is not intoxicating like THC and has been shown to be emotionally beneficial (i.e., anxiolytic), anti-inflammatory, and neuroprotective (Colizzi and Bhattacharyya, 2017). A recent study showed that, although CBD may minimize some of the negative side effects associated with THC and enhance its therapeutic efficacy (Bhattacharyya et al., 2012; Martin-Santos et al., 2012; Hindocha et al., 2015), the presence of CBD may increase THC metabolite plasma concentrations and subtly increase cognitive impairment compared with THC alone (Arkell et al., 2019). These results may have significant implications for PwMS using medicinal cannabis containing both THC and CBD. Estimates suggest that approximately 50% of PwMS in the US use cannabis for symptom relief, but only 48% of users possess a medical card (Kindred et al., 2017). Thus, there is widespread unlicensed, and often illegal, use of cannabis in MS, involving various formulations (e.g., THC : CBD ratios) and routes of administration (e.g., combustible and edible). Furthermore, the effects of different cannabis products are diverse, with pure THC products exhibiting very different effects than combination THC/CBD products. Therefore, it is not appropriate to transfer efficacy of one type of cannabis (e.g., high THC) to another (e.g., high CBD) or to group their effects together. Unfortunately, most reviews do not discriminate between different cannabis ratios and it is therefore improper to make any conclusions of the effectiveness of “cannabis” on neuropathic pain. Furthermore, these reviews also included clinical research studies with more controlled cannabis formulations, such as dronabinol or nabiximols. Although these are accepted as safe and effective long-term MS treatment options (Svendsen et al., 2004; Rog et al., 2005; Langford et al., 2013; Schimrigk et al., 2017), they are currently unavailable/illegal for PwMS in many countries outside Europe and are therefore not in the same usage paradigm self-prescribing cannabis users.

## MS-Related Pain

The incidence of pain in MS ranges from 29% to 86% (Stenager et al., 1995). Because there are many subjective variables, such as current psychological status, cognitive status, and environment that can affect a pain determination, there are no truly objective methods of measuring this highly subjective experience. Nevertheless, pain is associated with decreased health-related quality of life and impairments in physical and emotional functioning (Uritis et al., 2019). There are many kinds of MS-related pain, including neuropathic (continuous or intermittent), musculoskeletal, and mixed neuropathic and nonneuropathic pain (Ferraro et al., 2018). Many cannabis studies have focused on neuropathic pain in PwMS. However, limitations of these studies include the ways in which central neuropathic pain was diagnosed and defined, the subjective nature of pain assessments, and the difficulty in blinding patients because of the psychoactive side effects of some cannabinoid formulations. There are currently no uniform diagnostic criteria for defining central neuropathic pain. Most studies rely on a list of typical characteristics of neuropathic pain or rely on the physician’s diagnosis of a central pain condition after excluding nociceptive and peripheral neuropathy-related causes. Thus, making a determination as to whether cannabinoids are specifically treating the *central* component of neuropathic pain or neuropathic pain from *peripheral* etiologies becomes difficult.

Chronic pain syndromes differ in symptom and pathophysiological mechanisms. Some reviews on pain in MS have included all available controlled studies dealing with all forms of chronic pain. This approach is problematic for several reasons. First, chronic pain syndromes (neuropathic, nociceptive, and musculoskeletal) can differ considerably in their pathophysiological mechanisms and in their symptoms. Second, a pooled analysis of all pain syndromes without a subgroup analysis of pain syndromes/mechanisms provides clinicians and researchers insufficient orientation about which specific cannabis product (e.g., THC : CBD ratio) should be used for a given clinically defined pain syndrome, i.e., with a specific pain and sensory phenotype. A review of reviews (Nielsen et al., 2018), which included 11 reviews providing data from 32 studies, determined that cannabis was effective at treating MS-related pain. However, because this review did not distinguish between the various forms of pain, cannabis products, or routes of administration, it is methodologically incorrect to conclude any effectiveness of cannabis for neuropathic pain in PwMS. Neuropathic pain can have many dimensions, and cannabis might be effective for some dimensions of chronic pain, but not for others.

## Statistical Versus Clinical Significance

Another notable issue is that none of the reviews on cannabis and MS-related pain acknowledged the clinical relevance of statistical results. When evaluating the validity of a study, one must consider both the clinical and statistical significance of the findings. Studies that show statistically significant differences in two treatment options may lack practicality, and studies that claim clinical relevance may lack sufficient statistical significance to make a meaningful conclusion. Clinicians and researchers should not focus on small *p*-values alone to decide whether a treatment with cannabinoids is clinically useful; it is essential to consider the magnitude of treatment differences and the power of the study.

## Funding

Because of the required number of patients and study durations, clinical research studies on cannabis and multiple sclerosis are expensive. Therefore, it is not surprising that many of these studies are funded by pharmaceutical companies (Wade et al., 2004; Turcotte et al., 2015; Uritz et al., 2019). Unfortunately, missing publications of negative results for the investigated drug reported to the sponsor are still common (Speich et al., 2018). However, this is a problem in many other drug investigations and is not unique to cannabis-based medicine alone.

## Safety and Study Duration

The safety and potential long-term effects of cannabis products in PwMS have not been sufficiently evaluated. All of the MS studies so far were short term, ranging from 6 to 15 weeks. Long-term risks and rare, but severe, side effects cannot be captured in these short-duration trials. Another noteworthy observation is that there is no standardized way to compare the adverse effect profiles between the various cannabis formulations (i.e., of different THC : CBD ratios). In addition, it is difficult to assess whether certain adverse effects are dose dependent, which highlights another critical issue for future study.

THC levels and the THC : CBD ratio in cannabis have risen considerably in the USA and Europe in the last 2 decades, which may increase the potential harms from repeated use (Pacher et al., 2018). It is well established that cannabis, especially products with high THC content, negatively impairs cognition and executive function in healthy adults and may increase the risk of schizophrenic-like psychosis or other severe mental illnesses (Bhattacharyya et al., 2012; Bloomfield et al., 2019). This has significant implications for PwMS using medicinal cannabis because PwMS are already more likely to experience impaired cognition and executive dysfunction. Thus, the psychological effects of chronic cannabis use may further increase the risk of impairment in PwMS and lead to psychosis or severe mental illness. Currently, it is unknown how chronic cannabis use, of any THC : CBD ratio, impacts cognition and executive function in PwMS.

The 2016 report from the WHO on *The Health and Social Effects of Nonmedical Cannabis Use* notes the potency of cannabis as a likely factor in the rise in cannabinoid receptor 1 (CB1R)-mediated adverse cardiovascular effects, including death (Pacher et al., 2018). The cardiovascular effects of cannabis depend on several factors, including composition of the cannabis product (i.e., higher THC content in the plant and higher risk of CB1R-mediated cardiovascular effects). It is possible that cannabis products with higher relative amounts of CBD may be safer than products that have no or low CBD. However, this has yet to be determined since few laboratories also measure CBD in blood toxicology. To this end, from a cardiovascular standpoint, limiting or even eliminating THC from cannabis extracts used in the treatment of neuropathic pain in PwMS may be justified.

CBD is not risk-free and has both drug interaction and adverse event potential (Huestis et al., 2019). Because CBD is an effective anticonvulsant therapy, the FDA is concerned that it might cause suicidal ideation (White, 2019). On the contrary, a recent review (Iffland and Grotenhermen, 2017) substantiated and expanded the findings of an earlier review (Bergamaschi et al., 2011) about the favorable safety profile of CBD. However, various areas of CBD safety research still need to be extended and longer-term safety data are critically needed to fully appreciate CBD’s balance of benefit to harm.

## Interactions With Other Drugs

A variety of drugs are used to treat MS and its symptoms. These include disease-modifying drugs, corticosteroids, and others that target specific symptoms and health problems related to MS, like depression, bladder problems, spasticity, sexual problems, fatigue, pain, and emotional changes (National MS Society). The increasing use of medicinal cannabis for MS-related symptoms, such as pain and spasticity, can produce potential interactions with medications used for other symptomatic treatment. It is well known that cannabis, especially with higher THC content, induces sedation, impairs psychomotor performance, and increases blood pressure and heart rate. Pharmacodynamic interactions with other sedatives can potentiate the central effects, but sedation can be decreased by psychostimulants (Lindsey et al., 2012). These interactions are important to emphasize on medications that are either inductors or inhibitors of the isoenzymes that metabolize THC and CBD (Rong et al., 2017). Most of the known interactions can be found in the prescriber information for the approved medicinal cannabis products (dronabinol, nabilone, and nabiximols). However, this information is not available for the medicinal cannabis with different THC/CBD compositions available in other countries. Furthermore, there is a lack of data in humans about the possible interactions between synthetic cannabinoids and cannabis, but combining them may augment t psychopharmacological activity (have an additive effect) (Arellano et al., 2017).

## Conclusion

There is a “painful” parallel between cannabis and the past and present situation with opioids, where the short-term demonstration of efficacy on chronic pain led to the promotion and broad scale prescription of opioids in the absence of high-quality evidence. Thus, the lack of any clinically relevant beneficial effects of cannabis in most systematic reviews, the potential for clinically relevant side effects, and concerns about long-term risks should give clinicians pause before recommending the use of cannabis to treat neuropathic pain in PwMS.

## Author Contributions

The author confirms being the sole contributor of this work and approved it for publication.

## Conflict of Interest

The author declares that the research was conducted in the absence of any commercial or financial relationships that could be construed as a potential conflict of interest.
